# Remarkable Sympathetic Disturbance

**Published:** 1879-07

**Authors:** 


Dr. Spitzka, of the American Neurological Association, at its recent annual meeting held at New York, related the following case of remarkable sympathetic disturbance : A young man, a persistent masturbator, while engaged in the act suddenly heard a noise like the crack of a pistol in his head. From that time he became melancholic ; he noticed that one-half of his face, on the side where he had heard the sound, was hotter, perspired more than the other. In about three weeks a carbuncle appeared on the back of the neck, near the third cervical vertebra. After this, irregular yellow spots appeared on the affected side of the face, the parts atrophied, the hair became white and there were differences in the pupil. His mind became somewhat affected. It was suggested that the sound was probably due to the sudden vascular disturbance.

In a short discussion which followed, Dr. Spitzka referred to the fact that experiments and investigations showed the sympathetic system to be independent of mental disturbances, and that it could be greatly injured or diseased, and yet the mind not be at all affected.—*Medical Record.*

